# Shaping the future of abdominal and pelvic pain research with novel scientific and technological advances

**DOI:** 10.3389/fpain.2026.1738762

**Published:** 2026-02-26

**Authors:** Anna P. Malykhina

**Affiliations:** Division of Urology, Department of Surgery, University of Colorado Anschutz Medical Campus, Aurora, CO, United States

**Keywords:** abdominal pain, artificial intelligence methods, chronic pelvic pain, inflammaging, microphysiological systems

## Abstract

Abdominal and pelvic pain often originates from one or more visceral organs of the gastrointestinal, musculoskeletal (pelvic floor), urinary, or reproductive systems. Over the past decade, research efforts on abdominal and pelvic pain have advanced considerably, driven by the discovery of new molecular targets, signaling pathways, targeted genetic editing, the use of human tissues, and comprehensive multiomic analyses. Increasingly, the field prioritizes combinatorial and integrative studies that bridge human clinical research with relevant animal models to accelerate the development of novel therapies for affected patients. In addition to established areas of pain research—such as the modulatory role of the central nervous system in chronic pelvic pain (CPP), stress-induced visceral hypersensitivity, sex differences, brain-gut interactions, microbiome influences, comorbidities, and neuromodulation—new research directions continue to emerge. These include exploring the effects of inflammaging and immune regulation in transition from acute to CPP, applying microphysiological systems (MPS) in pain research studies, integrating multiomics analyses, and employing combined artificial intelligence (AI) approaches. This article presents current perspectives on cutting-edge scientific trends and technological innovations shaping the field of abdominal and pelvic pain research.

## Introduction

The field of abdominal and pelvic pain research has significantly evolved within the last decade with discoveries of novel molecular targets and signaling pathways due to increasing use of more precise clinical phenotyping, targeted genetic editing and complex multiomic approaches. Combinatorial and interactive investigations have become a top priority, with a tighter integration of clinical research on human subjects with appropriate animal models to facilitate the development of novel therapies for the affected patients. The latest basic science, translational and clinical research studies focused on elucidating pathophysiological mechanisms of chronic pelvic pain (CPP) conditions, identifying and matching clinical pain phenotypes with respective biomarkers, developing diagnostic tests, and validating new drugs to minimize symptoms and disease progression in patients with visceral pain (1). Abdominal or pelvic pain usually arises from one or more visceral organs belonging to gastrointestinal, musculoskeletal (pelvic floor), urinary or reproductive systems [*e*.*g*., urological chronic pelvic pain syndrome (UCPPS), vulvodynia, vestibular pain syndrome, dysmenorrhea, endometriosis, irritable bowel syndrome, inflammatory bowel disease, pelvic floor myofascial pain syndrome, and others]. In addition to well established topics in pain research (modulatory role of the CNS in the pathogenesis of CPP, stress-induced visceral hypersensitivity, sex differences, brain-gut interactions, the role of microbiome, co-morbidities, neuromodulation *etc.*), the increasing number of evolving research directions enters the field. They highlight the effects of inflammaging and immunoregulation in CPP signaling, the use of microphysiological systems (MPS) in pain research, integrated multiomics analyses and combined artificial intelligence (AI) approaches. This article outlines perspectives on the latest scientific directions and novel technological advances in the field of abdominal and pelvic pain research.

### Immunoregulation and inflammaging in CPP pathogenesis

Immunoregulation in CPP disorders is associated with immune cell infiltration to the affected organs, altered cytokine profiles and high prevalence of CPP comorbidities with known autoimmune diseases. Inflammaging is one of the CPP factors linked to a state of chronic, systemic, low-grade and mostly asymptomatic inflammation that develops with age. It affects immune and neuronal functions involving activation of pro-inflammatory macrophages, mast cells, and other cell types followed by the release of inflammatory molecules (2). One of the hypotheses for inflammaging is that macrophages are continually stimulated because of an increasing load of ‘molecular waste’ during aging leading to ongoing covert inflammation (3). Recent research efforts searched for the functional links between inflammaging, immunostimulation and the occurrence/persistence of CPP during aging (4). Increased levels of proinflammatory cytokines, immune imbalances leading to mucosal changes, and heightened visceral sensitivity have been reported in patients with gastrointestinal (GI) CPP pathologies (5). In the GI tract, inflammaging correlated with an increased density of mast cells that were closely aligned with intrinsic nerve terminals in the mucosa of the human distal gut (6). Mast cells and CD3^+^ *T* cells were also significantly elevated in colonic biopsies of patients with irritable bowel syndrome in comparison to healthy controls (7). An age-dependent loss in density of enteric glial cells was detected within myenteric ganglia and in the circular muscle of adult human colon suggesting potential changes in trophic support of intrinsic innervation (8). A recent study identified a mast cell-sensory neuron circuit that initiated bladder inflammation and simultaneously propagated neural hypersensitivity from the bladder to the colon in a murine model of interstitial cystitis/bladder pain syndrome (IC/BPS) (9). The data revealed anatomic heterogeneity of mast cells in relation to nociceptors in the bladder and their critical dependence on Mas-related G protein-coupled receptor B2 (MrgprB2) to promote visceral hypersensitivity and bladder-colon cross-sensitization. These studies highlighted close interactions between immune cells and sensory innervation as a contributing immunoregulatory mechanism that was also involved in the development of pelvic organ cross-sensitization (known to be a contributing factor in CPP) (10).

In the lower urinary tract, the role of immunoregulatory pathways has been most studied in the pathophysiology of IC/PBS (11, 12) with a confirmed up-regulation of several immune cell subtypes in the bladder tissue including mast cells, activated CD4+ *T* cells, and regulatory *T* cells (13). Several differences have been observed between the types of immune cells and cytokine profiles in ulcerative (with Hunner lesions) vs. non-ulcerative (no Hunner lesions) IC/BPS (14, 15). Ulcerative IC/BPS is characterized by chronic inflammation extending beyond Hunner lesions in 93% of the patients when compared to 8% of patients with non-ulcerative IC/BPS (16). In addition, cells of the adaptive immune system were elevated within Hunner`s lesions (15). On the contrary, non-ulcerative IC/BPS is associated with low levels of local inflammation but increased number of mast cells (17). Gene expression analysis of bladder biopsies from patients with non-ulcerative IC/BPS revealed an upregulation of leukotriene biosynthesis nociceptive pathway that is usually activated in inflammatory diseases and neuropathic pain (18). In comparison to the ulcerative type of IC/BPS, activation of the immune pathways was modest in non-ulcerative IC/BPS, limited to neutrophil chemotaxis and IFN-γ-mediated signaling (18). Experimentally induced IC/BPS symptoms were also suppressed by inhibition of NLRP3 inflammasome pathway via reduction in the number of CXCR3^+^ *T* cells, mast cells, and neutrophils in the urinary bladder (19) highlighting additional evidence for immunoregulatory pathogenesis in chronic pain disorders of the lower urinary tract.

Recent studies of sex-specific CPP disorders like chronic non-bacterial prostatitis/chronic pelvic pain syndrome (CP/CPPS) in men or localized provoked vulvodynia (LPV) in women have been providing more evidence for extended neuroimmune interactions as one of the contributing pathophysiological mechanisms. While CP/CPPS was previously thought to be a non-inflammatory disorder, autoimmune dysregulations in this pathology have been detected by several groups (20–23). The number of mast cells and expression levels of NGF, TrKA and PGP9.5 was increased in the prostates of mice with experimental autoimmune prostatitis and in prostatic tissues from patients with benign prostatic hyperplasia (24). Likewise, clinical studies and pre-clinical experimental models of vulvar pain established that altered inflammatory responses of tissue fibroblasts, mast cells, neutrophils and macrophages may be key to the development of chronic vulvar pain in LPV (25). Up to 8% of women after age of 40 develop LPV linked to an altered immune-mediated inflammatory response (26), immune deficiencies, single and/or multi-organ immune disorders, and allergy/atopy conditions (27). Vestibular biopsies from women with LPV contained abundance of immune cells including macrophages and increased numbers of nerve fibers (28). Excessive levels of epithelial NGF in LPV were associated with increased B cell infiltration and the presence of germinal centers (29). Elevated B lymphocytes and mature mucosal IgA-plasma cells were found in LPV tissues with B and T cells arranged into germinal centers representing local immune activation (30). Histological specimens of patients with vulvodynia also detected mast cells and the presence of a T-lymphocyte dominant inflammatory infiltrate in the majority of tested specimens (31). Overall, while sex-specific CPP disorders are usually focused on gender-related hormonal changes (estrogen/progesterone dependent sensitivity), neurological differences in the density of nociceptors and/or psychological factors (higher anxiety in women), neuro-immune crosstalk has been confirmed as one of the common pathophysiological mechanisms of visceral sensitization and chronic abdominal and pelvic pain in both sexes.

### The use of microphysiological systems in pain research

Microphysiological systems (MPS) include complex multicellular and multilayered three dimensional (3D) living models also known as “tissue-on-a-chip” or “organ-on-a-chip” (32). The MPS could be of animal or human origin and are designed to closely resemble physiologically relevant aspects of living tissues or organs with the goal of overcoming limitations of two-dimensional cell cultures. The development of human MPS is an emerging technology to mimic human physiology at the basic level with the potential to replace *in vivo* animal studies for evaluating safety and efficacy during the initial stages of drug and therapeutic development. MPS typically use 3D scaffolds with microfluidic compartments that model micro-scale units of multicellular organs (33), tissue interfaces (34), and multi-organ systems (35). The ability to embed micro-sensors to track oxygen (glucose, lactate, glutamate *etc.*) levels in the MPS provides an additional advantage for research studies (36). The database of currently available MPS was established by the University of Pittsburgh Drug Discovery Institute to aggregate and manage data from different laboratories and to provide reference and clinical data to evaluate and validate experimental MPS results (37).

Several MPS have been developed to model painful (nociceptive) signaling and to screen experimental compounds for their analgesic effects. One study modeled spinal cord dorsal horn by co-culturing peripheral and dorsal spinal neurons in a MPS to record surrogate signals for pain (38). Another group used cerebral organoids generated from induced pluripotent stem cells (iPSCs) of adult healthy individuals to confirm that opioid receptors are expressed in these 3D structures (39). Sensory neurons derived from iPSCs (iPSCs-SNs) have provided new avenues towards elucidating peripheral pathophysiological and pain-related mechanisms (40, 41). A comprehensive transcriptomic analysis of iPSCs-SNs confirmed the sensory neuron-like nature of these cells in addition to morphological evidence suggesting their pseudo-unipolar neuronal morphology (42).

Organoid models of the GI, urological and reproductive organs have been gaining more interest in the last decade. Remarkable progress has been made in the development of complex GI microtissues, especially with second generation models comprised of epithelial organoids co-cultured with non-epithelial cell types that can successfully reproduce intracellular interactions (43). The combinatorial approach of using growth factors, chemical gradients, and mechanical factors in stimulating cellular morphogenesis provides a diverse toolbox to guide the formation of specialized 3D GI microtissues (44). The critical step to advance available GI MPS for pain-related research would include integration of enterochromaffin cells that function as epithelial chemosensors with signal transducing enteric neurons. Emerging *in vitro* models have paired stem cells from human intestinal organoids with neural crest cells differentiated into neurons. When these cells are implanted *in vivo,* they form neuroglial structures similar to a myenteric and submucosal plexus (45).

The attempts to develop MPS from the urogenital system have been mostly focused on epithelial tissues. For example, uterine organoids were established from human and mouse endometrial epithelia (46), and were comprised of proliferating, secretory and progenitor endometrial cells (47). Recent studies of mouse (48) and human (49) urinary bladder epithelial organoids were used to study the mechanisms of urinary tract infections. Parab et al. (48) described mouse bladder epithelium-derived MPS as a scalable platform to model urothelial aging, and to demonstrate its utility for modeling uropathogenic *E. coli* (UPEC) infection. Zulk et al. (49) developed human urinary bladder organoids from bladder stem cells which recapitulated cellular diversity of the urothelium. Using bulk and single cell RNAseq, the group characterized the organoid responses to UPEC and phage exposure. Similarly, testicular organoids were derived from human primary testicular cells (50). Morphological evaluations revealed that they were formed by compact spherical structures composed of uncharacterized elongated fibroblast-like cells in the peripheral region. While epithelium-derived MPS are exciting first steps in the development of full-scale 3D organoids of the lower urinary and reproductive tracts, additional advancements to achieve adequate muscle layer presence, vascularization and innervation of the organoid tissue would be necessary to make them suitable for pelvic pain research.

### Combinatorial AI approaches and integrated analyses

The use of AI models has been increasingly entering the landscape of animal and human pain research. The range of AI-related technologies expands every day from classical machine learning (ML) to more complex multi-models providing an additional level of personalization to all domains of CPP research (51). The AI is no longer a complementary computational tool to traditional research methods but is necessary for complex analyses of large datasets and comparisons between research sites, treatments and predictions of the expected outcomes. The development of accurate and objective methods for pain detection and assessment including integration of pain sensors with AI technologies opened new possibilities for CPP research. Pain Intervention and Digital research (Pain-IDR) Program was launched in 2022 to test whether functional and pain status (an index of a patient`s physical, emotional, and social wellbeing) can be assessed passively (through a smartphone application) in older patients with chronic pain, thereby, combining outpatient clinical care with digital health research (52). The program successfully enrolled 77 patients with chronic pain and collected high-frequency ecological recordings of mobility, emotion and sociability (HERMES) to assess “moment-by-moment” quantification of a patient's functional state. Other models, like ChatGPT's Advanced Voice Mode were also proven useful to offer a personalized feedback to providers about effective communication with CPP patients to tailor their treatment (53). Communication in pain management is one of the known challenges in healthcare education and increasing applications of advanced voice interfaces such as ChatGPT in simulation-based educational programs enrich trainees` learning experience by practicing challenging communication scenarios (54).

One of the most transformative applications of AI-based technologies is its power to integrate. Multimodal AI tools can analyze combined imaging, histopathological and molecular signatures in addition to electronic health records to create detailed individualized patient representations. These digital phenotypes could offer specific predictions for chronic pain, immune responses and therapeutic outcomes in the affected patients’ cohorts. Large language models (LLM) further extend this integration by streamlining clinical workflows, facilitating patient communication, and rapidly assessing inclusion/exclusion criteria, endpoint validity and statistical feasibility (55). In urology research, applications of ML methods have been expanding from experimental prototypes to clinical settings. A recent study explored the use of ML models (CatBoost and XGBoost) to diagnose lower urinary tract symptoms (LUTS) by analyzing patient-reported outcomes, uroflowmetry measurements, and ultrasound data (56). The results revealed that while CatBoost was more sensitive and effective for initial screenings, XGBoost showed superior precision and specificity necessary for final diagnosis (56). More than 200 variables from the Neurogenic Bladder Research Group Spinal Cord Injury registry were used for a Decision Tree analysis (eCHAID technique) to identify parameters associated with the development of LUTS (57). The predictive capacity of this AI tool was found to be 57%, suggestive of somewhat limited capability to forecast future bladder symptoms (58). Additional AI models (DeepSeek-V3, DeepSeek-R1, OpenAI o3-mini, and OpenAI o3-mini high) were used to analyze urological questions in the areas of benign prostatic enlargement, urinary stones, infections, and respective guideline updates (57). The obtained results indicated that o3-mini high could serve as a quick second-opinion tool for outpatient counselling and protocol updates, whereas DeepSeek-R1 could provide a cost-effective alternative in resource-limited settings (57). The use of AI powerful analytics in women's health diagnostics has been gaining more attention due to its ability to identify patterns that have been challenging to decipher by conventional methods (59). Two hundred nine clinical features, including demographics, presenting symptoms, gynecologic/obstetric history, and physical exam findings, were analyzed as predictors of endometriosis by utilizing ML algorithms (60). The chosen ML methods successfully predicted endometriosis preoperatively, however, the applied approaches had several limitations. First, it was a retrospective case-control study without a follow-up validation of the model in a separate dataset. Second, the imbalance within the analyzed database with a predominant representation of endometriosis patients (83%) and only 16% of controls likely affected the algorithm training. Third, emesis emerged as the top positively predictive feature of endometriosis with cumulative influence score of 40 followed by crampy pain (score of 25). One of the explanations provided by the authors for this unexpected result was that ML models do not consider features independent of others, and each parameter is analyzed in conjunction with all other features (60). Fourth, the use of hormonal therapy/contraceptives was not taken into consideration, and the effects of hormones on pain symptoms are well established in the clinical setting. Overall, this study emphasized the importance of close correlation and co-development of ML algorithms with consideration of relevant biological processes and disease pathophysiology.

Artificial intelligence tools also transform the molecular landscape of pain research. AI-based techniques are currently applied to multidimensional transcriptomic and proteomic datasets, revealing novel therapeutic targets and signaling pathways. The immune profile of human IC/BPS was evaluated using three ML algorithms and revealed correlations between characteristic genes and immune cell subtypes in human urinary bladder specimens (13). The majority of immune cell subtypes were up-regulated in IC/BPS tissue, including mast cells, activated CD4^+^ *T* cells, and regulatory T cells that suppress immune responses (13). A similar ML-based approach was used for identification of mRNA signatures specific to non-ulcerative IC/BPS (61). mRNA signatures were analyzed by both supervised and unsupervised ML approaches to define IC/BPS classifiers for comprehending gene expression changes in the urinary bladder. An additional study used integrated analysis of differentially expressed genes (DEG), inflammation-related genes (IRG) and ML machine approach to find several diagnostic biomarkers of bladder fibrosis in patients with neurogenic bladders (NB) (62). First, using a rat model of NB, a combined analysis of DEG, IRG, and WGCNA was performed to identify NB-related hub genes. Next, four diagnostic biomarkers for NB (HIF-1α, IL-1β, CD40, and IL10RA) were pinned down using the MCODE plugin and ML methods. Finally, the expression levels of these biomarkers were tested in the bladder tissue of NB patients using immunohistochemical and Western blot analyses. The results revealed a significant up-regulation of HIF-1α, IL1-β, CD40, and IL10RA proteins in the NB specimens in comparison to healthy bladders (62). This recent work is a convincing example of a combinatorial translational approach of incorporating AI data mining with molecular and histological findings for biomarker discoveries.

In summary, integration of AI approaches into abdominal and pelvic research is not a simple technological advancement as it allows to move from observational findings to predictive modeling, from retrospective analysis to pathophysiological insights, and from scientist-exclusive interpretation to a new era of human–AI collaboration. As scientists, we should be aware of the common pitfalls associated with using AI in various aspects of pain research and have a clear understanding that the final interpretation of AI-revealed patterns and predictions should be always validated by human judgement.

## Conclusions

Multifactorial etiology of abdominal and pelvic pain and high level of CPP co-morbidities provide challenges in diagnosis, pathophysiology, and treatment of the affected patients. Knowledge gaps still exist around neuro-immune mechanisms and the role of inflammaging in the transition from acute to chronic pain conditions with increasing need for novel technological advances to analyze currently available large and complex biological datasets. From a drug discovery perspective, there is high demand for 3D *in vitro* systems that can reproduce anatomic distribution and biochemical mechanisms of different cell types forming a specific pelvic organ or tissue. Developed within the last decade MPS models attempt to recapitulate tissue morphology and function rather than single cell-type biology. In addition to evaluating the prognostic value of previously identified biomarkers for transition from acute to CPP, the use of AI-based methods provides a new level of data integration by analyzing multiple datasets and suggesting multimodal biosignatures of CPP phenotypes. While both MPS and AI approaches do have numerous limitations, their future advancements hold immense potential in opening additional avenues for CPP drug discovery and testing, and allow scientists to better model and understand neural, metabolic, and immune relationships in abdominal and pelvic pain research.

## Data Availability

The original contributions presented in the study are included in the article/Supplementary Material, further inquiries can be directed to the corresponding author.
